# Effectiveness and Safety of Risankizumab in Crohn Disease After Failure of a First Line of Advanced Therapy: A Long-Term, Real-Life Study

**DOI:** 10.1097/MJT.0000000000002171

**Published:** 2026-07-23

**Authors:** Antonio Tursi, Giammarco Mocci, Franco Scaldaferri, Daniele Napolitano, Federica Gaiani, Gianluigi De' Angelis, Davide Giuseppe Ribaldone, Eleonora Schillaci, Edoardo V. Savarino, Caterina D. E. Barba, Emanuele Bendia, Laura Bolognini, Daniele Balducci, Claudia Quatraccioni, Francesco Martini, Michele Montori, Giovanni Maconi, Giovanni Cataletti, Alessandro Massari, Elena Del Zanna, Francesco Costa, Linda Ceccarelli, Antonietta G. Gravina, Raffaele Pellegrino, Giorgia Bodini, Andrea Pasta, Manuela Marzo, Agnese Miranda, Antonio Cuomo, Laura Donnarumma, Giovanni Lombardi, Marta Patturelli, Mariaelena Serio, Antonella Scarcelli, Giuseppe Pranzo, Chiara Maria Palmisano, Rocco Spagnuolo, Francesco Luzza, Domenico Morano, Giuliana Vespere, Silvia Sedda, Leonardo D. E. Luca, Walter Elisei, Rita Monterubbianesi, Michela Di Fonzo, Libera Fanigliulo, Roberta Pica, Claudio Cassieri, Andrea Cocco, Elisa Stasi, Laurino Grossi, Lorenzo Bertani, Fabio Cortellini, Simona Piergallini, Patrizio Scarozza, Girolamo Bevevino, Giulia Zerboni, Federico Iacopini, Stefano Rodino', Ladislava Sebkova, Davide Checchin, Francesca Lamboglia, Francesco Ferrara, Nicola Della Valle, Raffaele Colucci, Francesco Bachetti, Domenico Catarella, Dario D'agostino, Elisabetta D. I. Bartolo, Ileana Luppino, Mario Erboso, Giulia Rocco, Carlotta Sacchi, Costantino Zampaletta, Giacomo Forti, Serafina Fiorella, Gabrio Bassotti, Elisabetta Antonelli, Caterina Mucherino, Elvira D'Antonio, Laura Montesano, Maurizio Giovannone, Giuseppina Vincoli, Alessandro Gigliozzi, Berardino D'Ascoli, Marina Rizzi, Pietro Capone, Giovanni Aragona, Patrizia Perazzo, Leonardo Allegretta, Paolo Tonti, Viviana Neve, Cristiano Pagnini, Antonio Grandolfo, Francesco Gatti, Antongiulio Bucci, Massimiliano Rizzi, Marcello Picchio, Francesca Maria Onidi, Paolo Usai Satta, Alfredo Papa

**Affiliations:** 1 Territorial Gastroenterology Service, ASL BAT, Andria, Italy; 2 Department of Medical and Surgical Sciences, Catholic University, School of Medicine, Rome, Italy; 3 Division of Gastroenterology, AORN “Brotzu” Hospital, Cagliari, Italy; 4 Digestive Diseases Center (CEMAD), Department of Medical and Surgical Sciences, Policlinico Universitario “A. Gemelli” Foundation, IRCCS, Rome, Italy; 5 Catholic University, School of Medicine, Rome, Italy; 6 Gastroenterology and Endoscopy Unit, Department of Medicine and Surgery, University of Parma, Parma, Italy; 7 Department of Medical Sciences, Division of Gastroenterology, University of Turin, IBD Unit, A.O.U. Città della Salute e della Scienza di Torino, Turin, Italy; 8 Gastroenterology Unit, Azienda Ospedale-Università di Padova (AOUP), Padua, Italy; 9 Division of Digestive Diseases, Digestive Endoscopy and Inflammatory Bowel Diseases, A.O. “Ospedali Riuniti”, Ancona, Italy; 10 Gastroenterology Unit, Department of Biomedical and Clinical Sciences, “L. Sacco” University Hospital, Milan, Italy; 11 IBD Unit, Department of General Surgery and Gastroenterology, Pisa University Hospital, Pisa, Italy; 12 Department of Precision Medicine, Hepatogastroenterology and Digestive Endoscopy Unit, University of Campania “Luigi Vanvitelli”, Naples, Italy; 13 Department of Internal Medicine and Medical Specialities, Division of Gastroenterology, IRCCS “San Martino” Hospital, University of Genoa, Genoa, Italy; 14 Division of Gastroenterology, “Veris-Delli Ponti” Hospital, Scorrano, Italy; 15 Division of Gastroenterology, “Monaldi” Hospital, AORN “Dei Colli”, Napoli, Italy; 16 Division of Gastroenterology, “Umberto I” Hospital, Nocera Inferiore, Italy; 17 Division of Gastroenterology, AORN “Cardarelli”, Naples, Italy; 18 Division of Gastroenterology, “San Salvatore” Hospital, Pesaro, Italy; 19 Ambulatory for IBD Treatment, “Valle D’Itria” Hospital, Martina Franca, Italy; 20 Department of Health Science, University of Catanzaro, Catanzaro, Italy; 21 Division of Gastroenterology, “Ospedale del Mare”, ASL Na1, Naples, Italy; 22 Division of Gastroenterology, A.O. “S. Camillo-Folanini”, Rome, Italy; 23 Division of Gastroenterology, “S.S. Annunziata” Hospital, Taranto, Italy; 24 Division of Gastroenterology, IBD Unit, “S. Pertini” Hospital, Rome, Italy; 25 Division of Gastroenterology, “Vito Fazzi” Hospital, Lecce, Italy; 26 Gastroenterology Unit, “Spirito Santo” Hospital, “G. d'Annunzio” University, Pescara, Italy; 27 Division of Gastroenterology, “Felice Lotti” Hospital, Azienda USL Toscana Nord Ovest, Pontedera, Italy; 28 Division of Gastroenterology, “Infermi” Hospital, Rimini, Italy; 29 Division of Gastroenterology, IBD Unit, “A. Murri” Hospital, Fermo, Italy; 30 Division of Gastroenterology, “Ospedale dei Castelli”, Ariccia, Italy; 31 Division of Gastroenterology, “Ciaccio-Pugliese” Hospital, Catanzaro, Italy; 32 Division of Gastroenterology,” S Giovanni e Paolo” Hospital, Mestre – Venezia, Italy; 33 Division of Gastroenterology, A.O. “Ospedali Riuniti”, Foggia, Italy; 34 Digestive Endoscopy Unit, “San Matteo degli Infermi” Hospital, Spoleto, Italy; 35 Division of Gastroenterology, ARNAS “Garibaldi”, Catania, Italy; 36 Division of Gastroenterology, “Annunziata” Hospital, Cosenza, Italy; 37 Division of Gastroenterology, “Belcolle” Hospital, Viterbo, Italy; 38 Division of Digestive Endoscopy, “S. Maria Goretti” Hospital, Latina, Italy; 39 Division of Gastroenterology, “P. Pio” Hospital, Vasto, Italy; 40 Gastroenterology & Hepatology Section, Department of Medicine & Surgery, University of Perugia, Italy; 41 Division of Gastroenterology, Azienda Ospedaliera “S. Anna e S. Sebastiano”, Caserta, Italy; 42 Division of Gastroenterology, “San Camillo Dè Lellis” Hospital, Rieti, Italy; 43 Division of Gastroenterology, “Madonna delle Grazie” Hospital, Matera, Italy; 44 Division of Gastroenterology, “T. Maresca” Hospital, Torre del Greco, Italy; 45 Division of Gastroenterology, “Guglielmo da Saliceto” Hospital, Piacenza, Italy; 46 Division of Gastroenterology, “Santa Caterina Novella” Hospital, Galatina, Italy; 47 Division of Gastroenterology, “A. Perrino” Hospital, Brindisi, Italy; 48 Division of Gastroenterology, “S. Giovanni – Addolorata” Hospital, Rome, Italy; 49 Division of Gastroenterology, “S. Paolo” Hospital, Italy; 50 Division of Digestive Endoscopy, “A. Perinei” Hospital, Altamura, Italy; 51 Division of General Surgery, “P. Colombo” Hospital, ASL Roma 6, Velletri, Italy

**Keywords:** risankizumab, real-world, clinical remission, Crohn's disease

## Abstract

**Background:**

Risankizumab (RZB) is an anti-interleukin 23 (anti-IL-23) approved for the treatment of Crohn disease (CD).

**Study question:**

We aimed to evaluate the effectiveness and safety of RZB in the treatment of CD in real-world settings.

**Study design:**

We performed a retrospective review of a multicentre consortium of patients with CD treated with RZB.

**Measures and Outcomes:**

Coprimary outcomes were clinical remission at week 12, 24, and 52 (Harvey–Bradshaw Index score of ≤4), and safety. Secondary outcomes included steroid-free clinical remission, clinical response, and endoscopic remission at 52 weeks.

**Results:**

A total of 487 patients were included. The median follow-up was 24 (interquartile range: 16–38) weeks. A total of 372 (76.4%) patients achieved clinical remission at maximal follow-up. According to the treatment line in which RZB was administered, clinical remission occurred in 115/134 (85.8%) patients on second-line therapy, 112/153 (73.2%) on third-line therapy, and 145/200 (72.5%) on fourth-line therapy, with a significant difference (*P* = 0.010). Adverse events occurred in 60 patients (12.6%) during follow-up; most of them were mild (50/60, 83.3%). Regarding the secondary outcomes: steroid-free clinical remission was achieved in 342 (71.8%) patients, and clinical response was observed in 443 (91.0%) patients. Six (1.2%) patients underwent surgery during follow-up. Mucosal healing was achieved in 34/67 (50.7%) patients.

**Conclusions:**

In this extensive, real-world study, RZB was effective and well tolerated in an advanced-therapy–exposed population of patients with CD and associated with favorable clinical and endoscopic outcomes.

**KEY MESSAGES**

- In the real world, Risankizumab (RZB), an anti-Interleukin 23 (IL-23) monoclonal antibody, reaches clinical remission up to around 80% in patients with Crohn disease (CD). However, current data arise mainly from short-term or small sample–size studies.
- Assessing a large population of patients with CD, treated with RZB after failure of a first treatment with advanced therapy up to 52 weeks, the clinical remission rate varies according to RZB use: it occurred in 115/134 (85.8%) patients on second-line treatment, 112/153 (73.2%) on third-line therapy, and 145/200 (72.5%) on fourth-line therapy.
- Clinical remission at 12 weeks predicted clinical remission at the end of follow-up; age >40 years and being on the third/fourth line of treatment were negative predictors for clinical remission.
- Adverse events occurred in 60 patients (12.6%) during follow-up; most of them were mild and did not require stopping treatment.

## INTRODUCTION

Risankizumab (RZB) is a humanized monoclonal immunoglobulin antibody which selectively inhibits interleukin (IL)-23 through high-affinity binding of its p19 subunit.^1^ IL-23 is hypothesized to play a critical role in the pathogenesis and propagation of inflammation in Crohn disease (CD).^2–4^ Patients with inflammatory bowel disease (IBD) have been shown to have higher circulating levels of IL-23 as compared with healthy controls, with the highest levels identified in CD as compared with ulcerative colitis.^2^ Genome-wide association studies have also identified polymorphisms in the gene coding for IL-23 receptor to be associated with the risk of incident CD.^2^

The phase 3 trials ADVANCE,^5^ MOTIVATE,^5^ and FORTIFY^6^ demonstrated that RZB was efficacious in both inducing and maintaining remission in patients with moderate-to-severe active CD, meeting the coprimary end points of clinical remission and endoscopic response. Given the strict inclusion criteria and tightly controlled environment of clinical trials, results cannot always be extrapolated to the broader population, making it paramount to evaluate RZB's effectiveness in real-world (RW) clinical settings to enhance generalizability.

In addition to needing RW effectiveness data, we must also augment our understanding of how RZB performs based on previous therapy exposure and other clinical factors. Consistent with previous data from biologic therapies, bio-naive individuals in the phase 3 pivotal trials experienced superior efficacy compared with the bio-exposed population.^5,6^ However, of particular interest to RZB is how it performs in individuals with previous exposure to ustekinumab (UST), another inhibitor that targets both IL-12 and IL-23 through the p40 subunit. Data suggest that blockade of IL-23 may play a more central role in modulating inflammatory responses than IL-12 blockade, and recent results from the head-to-head SEQUENCE trial indicate that RZB was superior in achieving both clinical and endoscopic end points compared with UST.^4,7^ The efficacy of RZB in UST-treated patients could be thoroughly evaluated in the pivotal phase 3 trials because of low numbers of UST-treated patients (enrollment was capped at 20% for each trial) and the fact that nearly all UST-treated patients had also been exposed to a tumor-necrosis factor (TNF)-α inhibitor. Given the expanding armamentarium of advanced therapies in CD, we must understand predictors of therapeutic response to better position these agents within our treatment paradigm.

For these reasons, we conducted a multicenter retrospective cohort study to assess the effectiveness and safety of RZB, and predictors of treatment response, in a RW population of patients with CD who failed other advanced therapies.

## METHODS

We conducted a retrospective, observational, multicenter study that included biologic-experienced CD outpatients who had failed at least 1 treatment with 1 or more of the following drugs: anti-TNFα antibodies (infliximab and/or adalimumab), UST, vedolizumab, or upadacitinib, and who were treated with RZB across 44 Italian IBD centers.

Men and women at least 18 years of age with a CD diagnosis established according to standard endoscopic, radiologic, and/or histological criteria were considered eligible.^8^ Exclusion criteria: patients presenting with a diagnosis of unclassified IBD, intestinal strictures or complications accompanied by surgical indications, a stoma, extensive bowel resection (≥2 bowel surgical resections and/or 50 cm of ileal resection),^9^ intestinal failure, and those receiving dual advanced therapy (ie, simultaneously using RZB plus another biologic agent or a small molecule drug).

In addition, we excluded patients treated with RZB as first-line advanced therapy, because at the moment in Italy, RZB can be authorized as first-line therapy for patients with CD only in cases of particular clinical conditions or contraindications to other advanced therapies.

A common database was created to collect demographic and clinical data. At baseline, we collected the following data: sex, age at diagnosis, current smoking status, presence of comorbidities, previous appendectomy, previous surgery for CD, the extension of the disease according to the Montreal classification, disease duration, previous immunosuppressive and biologic therapies (anti-TNFα agents, vedolizumab, UST, and upadacitinib), concomitant medications, fecal calprotectin (FC), C-reactive protein (PCR), erythrocyte sedimentation rate (ESR), Harvey–Bradshaw Index (HBI), Simple Endoscopic Score for CD (SES-CD), and Rutgeerts score for endoscopy (for patients with previous surgery).

We conducted the study in accordance with the clinical practice guidelines and the principles of the Declaration of Helsinki. All patients provided written informed consent before undergoing endoscopy and UST treatment. The reference centre (Brotzu Hospital, Cagliari, Italy, PROT. PG/2020/9414, 29 April 2020) obtained ethics committee approval for this retrospective study, which the other centers subsequently approved as well.

### Study treatment

All patients were treated uniformly with a label-based dosing schedule of 3 doses of intravenous (IV) RZB 600 mg at weeks 0, 4, and 8, followed by subcutaneous (sc) RZB 360 mg at week 12, then every 8 weeks thereafter.

The investigators were left to determine whether treatment discontinuation was needed. They were also left to judge the concomitant use of oral and topical aminosalicylates, steroids, and/or immunosuppressants.

### Clinical assessment at baseline and during the follow-up

The Montreal classification was used to assess disease extension,^10^ whereas the HBI score was used to evaluate disease activity.^11^ All the patients included in the study expressed active disease at the time of RZB enrollment, defined as an HBI score ≥5 points,^11^ despite concomitant treatment. Patients were clinically evaluated, along with CRP and FC levels, at entry and then again at 4, 8, 12, 16, 24, and 52 weeks, or on loss of clinical response.

### Endoscopy assessment at baseline and during the follow-up

All patients underwent a colonoscopy before starting RZB treatment per standard protocol at the participating centers. After 1 year of follow-up or at another time, depending on the patient's clinical history and the clinician's discretion, an ileo-colonoscopy with or without biopsies was performed as clinically indicated to monitor disease activity or for cancer surveillance. The SES-CD and Rutgeerts score (for patients with previous surgery) were used to assess endoscopic severity.^12–14^ Central reading for the assessment of the endoscopic activity was not performed.

### Outcomes

- The primary outcome was to assess the effectiveness of RZB for clinical remission (CR) (HBI of <5) after induction (week 12), after 24 weeks, and after 52 weeks. We assessed the effectiveness of RZB after failure of 1 (second-line), 2 (third-line), or 3 (fourth-line) previous treatments with advanced therapies.
- The coprimary outcome was the safety of RZB, defined as the absence of adverse events (AEs) during the follow-up. The AEs were subdivided into early events (occurring during the infusions) and late events (occurring at least 1 week after the infusion/injection) and graded as mild (not requiring treatment interruption) or severe (requiring treatment interruption).

In addition, this study provided several secondary outcomes:

- Steroid-free clinical remission (SFCR) during the study;
- Clinical response, defined as a reduction of HBI score of at least 2 points during the follow-up;
- Occurrence of any surgical procedure related to the disease in CD;
- Mucosal healing, defined as SES-CD ≤ 2 in patients with CD; and
- CRP and FC variations during follow-up.

### Statistical analysis

The data are presented using descriptive statistics. Continuous variables are expressed as the median and interquartile range (IQR); dichotomous or ordinal variables are presented as counts (percentages) of patients.

Clinical remission was considered the primary end point. The predictive value of the clinical parameters was evaluated using time-to-event methods for censored observations, given the varying follow-up durations. Follow-up times were calculated from the date of diagnosis to the date of event or censorship. The time-to-event analysis used Kaplan–Meier estimates to plot cumulative incidence curves, which were compared using the log-rank test and univariate and multivariate Cox proportional hazards models for the prognostic variables. The hazard ratios are presented with the 95% confidence intervals (CIs) and *P*-values. A ratio higher than unity implies that an event has a higher probability than that of the reference group. The Friedman test was used to assess changes in CRP and FC levels over the follow-up period. *P* < 0.05 was considered statistically significant.

## RESULTS

### Baseline characteristics

A total of 497 patients were treated with RZB at the enrolling centers; 10 patients were excluded from the analysis because they had not completed the induction period. Four hundred and eighty-seven patients were therefore included in the study, and the median follow-up was 24 (16–38) weeks, with 476 (95.8%) patients assessed at 12-week follow-up, 254 (51.1%) patients at 24-week follow-up, and 111 (22.3%) patients at 52-week follow-up. Table 1 shows the characteristics of the study group. In most patients, CD was localized in both the ileal and ileocolonic tracts, and stricturing disease was the most common phenotype (47.4%).

**Table 1.** Demographic and clinical characteristics of the Crohn disease patients at enrollment.

Characteristics	N (%)*
Sex, male	296 (60.8)
Age, median (IQR), yr	50 (34–60)
CD duration, median (IQR), yr	12 (6–22)
Presence of comorbidities	304 (62.4)
Previous surgery	284 (58.3)
Previous appendectomy	114 (23.4)
Smoking	124 (25.7)
Previous therapies	
Mesalazine	300 (61.6)
Steroids	425 (87.3)
Thiopurine	163 (33.5)
Methotrexate	56 (11.5)
Anti-TNF	437 (89.7)
Vedolizumab	139 (28.5)
Ustekinumab	264 (54.2)
Anti-JAK	56 (11.5)
Location	
Terminal Ileum	144 (29.6)
Colon	92 (18.9)
Ileo-colon	244 (50.1)
Upper gastrointestinal tract	7 (1.4)
Behavior	
Inflammatory	163 (33.5)
Structuring	231 (47.4)
Penetrating	93 (19.1)
Perianal disease	75 (15.4)
Line of therapy	
Second	134 (27.5)
Third	153 (31.4)
Fourth	200 (41.1)
Concomitant therapies	
No	282 (57.9)
Steroids	98 (20.1)
Mesalazine	78 (16.0)
Thiopurines	19 (3.9)
Methotrexate	8 (1.6)
Isoniazid	2 (0.4)
HBI, median (IQR)	6 (6–10)
SES-CD, median (IQR)	8 (6–12)
Rutgeerts score	3 (2–4)
Erythrocyte sedimentation rate, mm/h	28.0 (15.0–46.5)
C-reactive protein, mg/L	3.0 (0.9–10.0)
Fecal calprotectin, μg/g	363.0 (180.5–800.0)

* Unless otherwise specified.

TNF, tumor necrosis factor; JAK, Janus Kinase; HBI, Harvey–Bradshaw Index; SES-CD, Simple Endoscopic Score–Crohn Disease.

Regarding previous biologic therapies, most of the patients were already treated with an anti-TNF agent (89.7%) and UST (54.2%); about the line of treatment, 134 (27.5%) patients were treated with RZB as second line, 153 (31.4%) were treated as third line, and 200 (41.1%) were treated as fourth line of treatment.

### Primary outcomes

**FIGURE 1.**
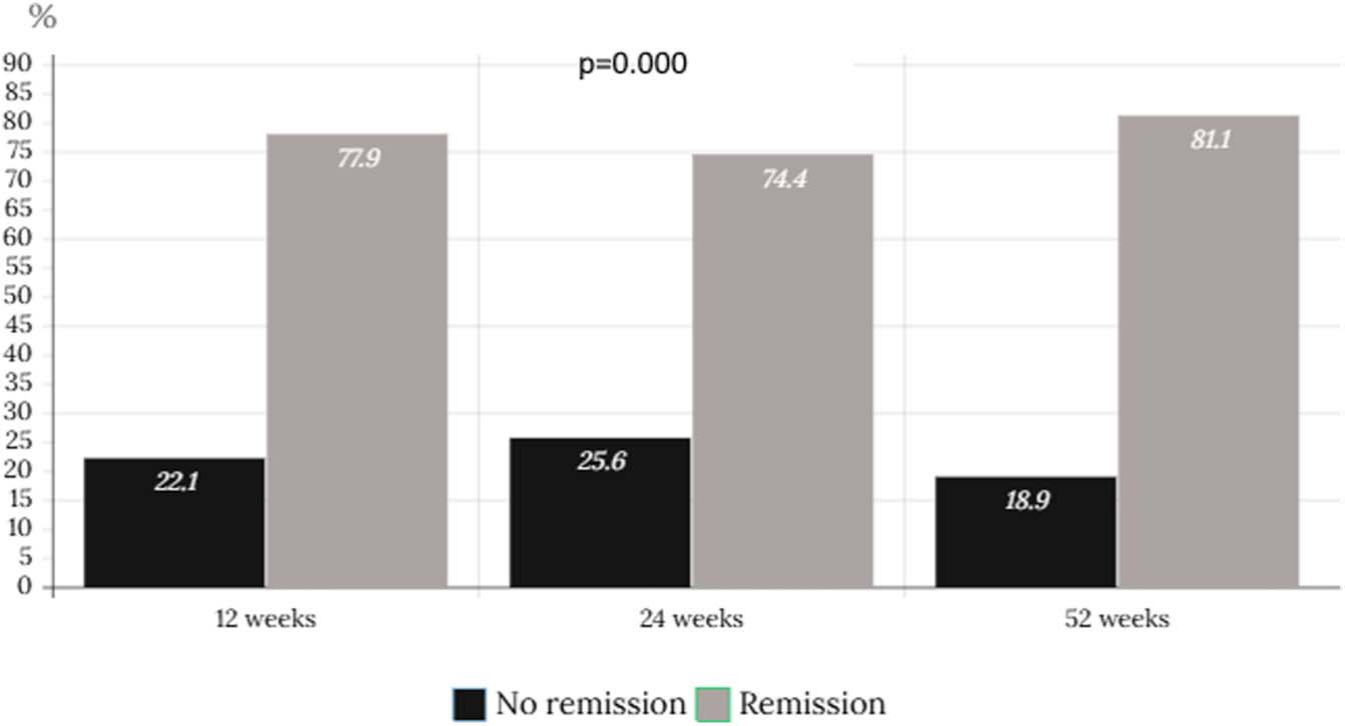
Clinical remission through the follow-up.

CR at maximal follow-up was observed in 372 (76.4%) patients. After induction (12 weeks), clinical remission was achieved in 371/476 (77.9%) patients; in 189/254 (74.4%) patients at 24-week and 90/111 (81.1%) patients at 52-week follow-up, respectively (*P* = 0.000; Figure 1).

**FIGURE 2.**
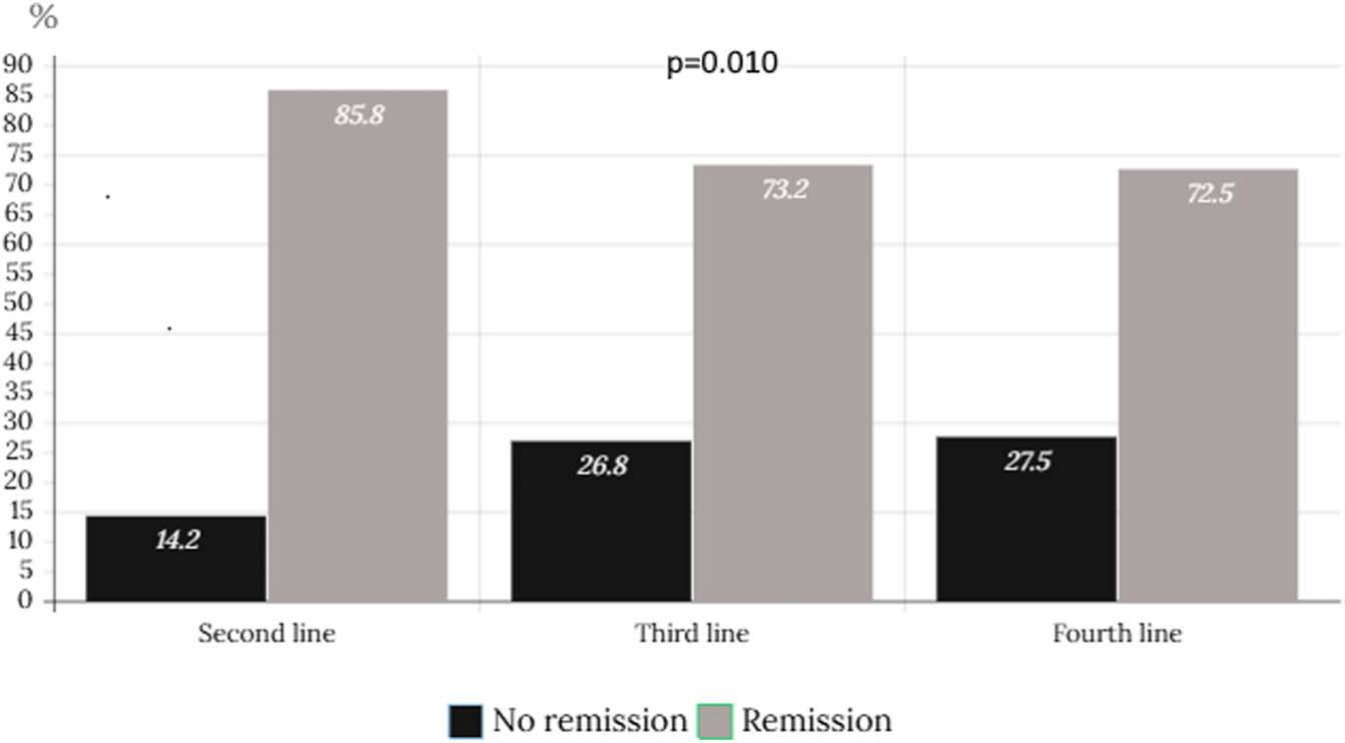
Clinical remission at maximal follow-up according to the line of treatment.

According to the line of treatment, CR at maximal follow-up showed a significant difference: it occurred in 115/134 (85.8%) patients with RZB as second line therapy, in 112/153 (73.2%) patients as third line therapy, and in 145/200 (72.5%) patients as fourth line therapy (*P* = 0.010; Figure 2).

Factors predicting CR at 12 weeks were analyzed using both univariate and multivariate analyses, and the results are reported in Table 2. At univariate analysis negative predictor of CR were age >40 years [odds ratio (OR) = 0.569 (95% CI, 0.348–0.929), *P* = 0.024], previous surgery [OR = 0.631 (95% CI, 0.401–0.994) *P* = 0.047], HBI > 10 [OR = 0.172 (95% CI, 0.104–0286), P=<0.000], SES-CD > 6 [OR = 0.631 (95% CI, 0.401–0.994), *P* = 0.047], Rutgeers score >3 [OR = 0.379 (95% CI, 0.198–0.723), *P* = 0.003], and third [OR = 0.389 (95% CI, 0.199–0.757), *P* = 0.005] and fourth [OR = 0.356 (95% CI, 0.188–0.675), *P* = 0.001] line of therapy compared with second line of treatment. At multivariate analysis negative predictor of CR were age >40 years [OR = 0.252 (95% CI, 0.085–0.938), *P* = 0.039], Rutgeers score >3 [OR = 0.281 (95% CI, 0.100–0.789), *P* = 0.016], and third [OR = 0.160 (95% CI, 0.033–0.776), *P* = 0.005] and fourth [OR = 0.224 (95% CI, 0.053–0.958), *P* = 0.044] line of therapy with respect to second line of treatment.

**Table 2.** Predictors of failure of remission at 12-week follow-up.

Variable	Total	No remission	Remission	Univariate analysis				Multivariate analysis			
	476	105 (22.1)	371 (77.9)	OR	95% CI	*P*		OR	95% CI	*P*	
Sex											
Male	289 (60.7)	61 (21.1)	228 (78.9)				Ref.				
Female	187 (39.3)	44 (23.5)	143 (76.5)	0.869	0.560–1.351	0.534					
Age											
≤40 yrs	162 (34.0)	26 (16.0)	136 (84.0)				Ref.				Ref.
>40 yrs	314 (66.0)	79 (25.0)	235 (74.8)	0.569	0.348–0.929	0.024		0.252	0.085–0.938	0.039	
Disease duration											
≤10 yrs	211 (44.3)	40 (19.0)	171 (81.0)				Ref.				
>10 yrs	265 (55.7)	65 (24.5)	200 (75.5)	0.720	0.462–1.122	0.146					
Smoke											
No	356 (74.8)	78 (21.9)	278 (78.1)				Ref.				
Yes	120 (25.2)	27 (22.5)	93 (77.5)	0.966	0.588–1.588	0.893					
Comorbidities											
No	172 (36.1)	41 (23.8)	131 (76.2)				Ref.				
Yes	304 (63.9)	64 (21.1)	240 (78.9)	1.174	0.751–1.834	0.482					
Previous surgery											
No	199 (41.8)	35 (17.6)	164 (82.4)				Ref.				Ref.
Yes	277 (58.2)	70 (25.3)	207 (74.7)	0.631	0.401–0.994	0.047		0.531	0.321–1.090	0.059	
Previous appendectomy											
No	364 (76.5)	80 (22.0)	284 (78.0)				Ref.				
Yes	112 (23.5)	25 (22.3)	87 (77.7)	0.980	0.589–1.631	0.939					
Disease location											
Terminal ileum	143 (30.0)	26 (18.2)	117 (81.8)				Ref				
Colon	88 (18.5)	25 (28.4)	63 (71.6)	0.560	0.299–1.050	0.070					
Ileo-colon	238 (50.0)	52 (21.8)	186 (78.2)	0.795	0.470–1.343	0.391					
Upper gastrointestinal tract	7 (1.5)	2 (28.6)	5 (71.4)	0.556	0.102–3.023	0.496					
Disease behavior											
Inflammatory	160 (33.6)	27 (16.9)	133 (83.1)				Ref.				
Stenosing	226 (47.5)	54 (23.9)	172 (76.1)	0.647	0.387–1.081	0.097					
Fistulizing	90 (18.9)	24 (26.7)	66 (73.3)	0.558	0.299–1.042	0.067					
Perianal disease											
No	401 (84.2)	95 (23.7)	306 (76.3)				Ref.				
Yes	75 (15.8)	10 (13.3)	65 (86.7)	2.018	0.998–4.082	0.051					
HBI											
≤10	391 (82.1)	61 (15.6)	330 (84.4)								Ref.
>10	85 (17.9)	44 (51.8)	41 (48.2)	0.172	0.104–0.286	<0.000		0.375	0.133–1.054	0.063	
SES-CD											
≤6	82 (29.0)	13 (15.9)	69 (84.1)				Ref.				Ref.
>6	201 (71.0)	55 (27.4)	146 (72.6)	0.500	0.256–0.976	0.042		0.701	0.191–2.568	0.592	
Rutgeers											
≤3	107 (54.3)	20 (18.7)	87 (81.3)				Ref.				Ref.
>3	90 (45.7)	34 (37.8)	56 (62.2)	0.379	0.198–0.723	0.003		0.281	0.100–0.789	0.016	
Naive to anti-TNFα											
Yes	49 (10.3)	7 (14.3)	42 (85.7)				Ref.				
No	427 (89.7)	98 (23.0)	329 (77.0)	0.559	0.247–1.285	0.171					
Naive to vedolizumab											
Yes	340 (71.4)	63 (18.5)	227 (81.5)				Ref.				Ref.
No	136 (28.6)	42 (30.9)	94 (69.1)	0.509	0.323–0.802	0.004		0.593	0.172–2.046	0.408	
Naive to ustekinumab											
Yes	218 (45.8)	39 (17.9)	179 (82.1)				Ref.				Ref.
No	258 (71.4)	66 (25.6)	192 (74.4)	0.634	0.406–0.989	0.045		0.460	0.129–1.741	0.253	
Naive to anti-JAK											
Yes	420 (88.2)	89 (21.2)	331 (78.8)				Ref.				Ref.
No	56 (11.8)	16 (28.6)	40 (71.4)	0.672	0.360–1.256	0.213					
Therapy line											
Second	123 (25.8)	14 (11.4)	109 (88.6)				Ref.				Ref.
Third	153 (32.1)	38 (24.8)	115 (75.2)	0.389	0.199–0.757	0.005		0.160	0.033–0.776	0.023	
Fourth	200 (42.0)	53 (26.5)	147 (73.5)	0.356	0.188–0.675	0.001		0.224	0.053–0.958	0.044	
Erythrocyte sedimentation rate, mm/h											
≤30	90 (54.5)	25 (27.8)	65 (72.2)				Ref.				
>30	75 (45.5)	24 (32.2)	51 (68.0)	0.817	0.418–1.596	0.555					
C-reactive protein, mg/L											
≤3	223 (49.8)	49 (22.0)	174 (78.0)				Ref.				
>3	225 (50.2)	51 (22.7)	174 (77.3)	0.961	0.616–1.499	0.860					
Fecal calprotectin, μg/g											
≤400	181 (53.1)	38 (21.0)	143 (79.0)				Ref.				
>400	160 (46.9)	36 (22.5)	124 (77.5)	0.915	0.547–1.532	0.736					

Values are expressed as number (percentage) of patients, unless otherwise specified.

Ref., reference HBI; Harvey–Bradshaw Index; SES-CD, Simple Endoscopic Score–Crohn Disease; TNF, tumor necrosis factor; JAK, Janus Kinase.

Regarding the safety profile, AEs occurred in 60 patients (12.6%) during follow-up; most of these (36 events, 60%) were early AEs. AEs were mild in 50 (83.3%) cases and severe in the remaining 10 (16.7%) patients (Table 3). Overall, most AEs were infectious, mainly affecting the respiratory tract, and occurred as early and late AEs. Two malignancies were recorded during the follow-up. The first was a 54-year-old man with a CD history of 19 years, and already treated with infliximab and adalimumab, who developed an osteosarcoma of the right humerus at the 24th week of treatment; the second was a 70-year-old woman with several comorbidities (hyperaldosteronism, renal insufficiency, and Ehlers–Danlos syndrome), who developed an intestinal neuroendocrine tumor of the ileum 20 weeks after starting therapy with RZB.

**Table 3.** Adverse events recorded during the study.

	Early	Late
Total AE	24	36
Mild-to-moderate AE	19	31
Bronchitis	4	7
Hypertension	1	—
Cholangitis	1	—
Insomnia	1	—
Intestinal infection	3	3
Skin lesions	3	5
Headache	1	1
Joint pain	4	4
Sinusitis/faringitis	1	2
Depressive syndrome	—	1
Fever	—	2
Abdominal pain	—	4
Pelvic abscess	—	1
Diarrhea	—	1
Severe AE	5	5
Lung infection	1	—
Infection (leishmania)	1	—
Renal failure/Hypotension	1	—
Allergy	2	—
Abdominal abscess	—	1
Herpes-zoster infection	—	1
Skin lesions	—	1
Osteosarcoma	—	1
NET	—	1

### Secondary outcomes

SFCR was achieved in 342 (71.8%) patients. Clinical response was observed in 443 (91.0%) patients. Surgical procedures related to CD were necessary in 6 cases (1.2%) during follow-up. Mucosal healing was achieved in 34/67 (50.7%) patients.

**FIGURE 3.**
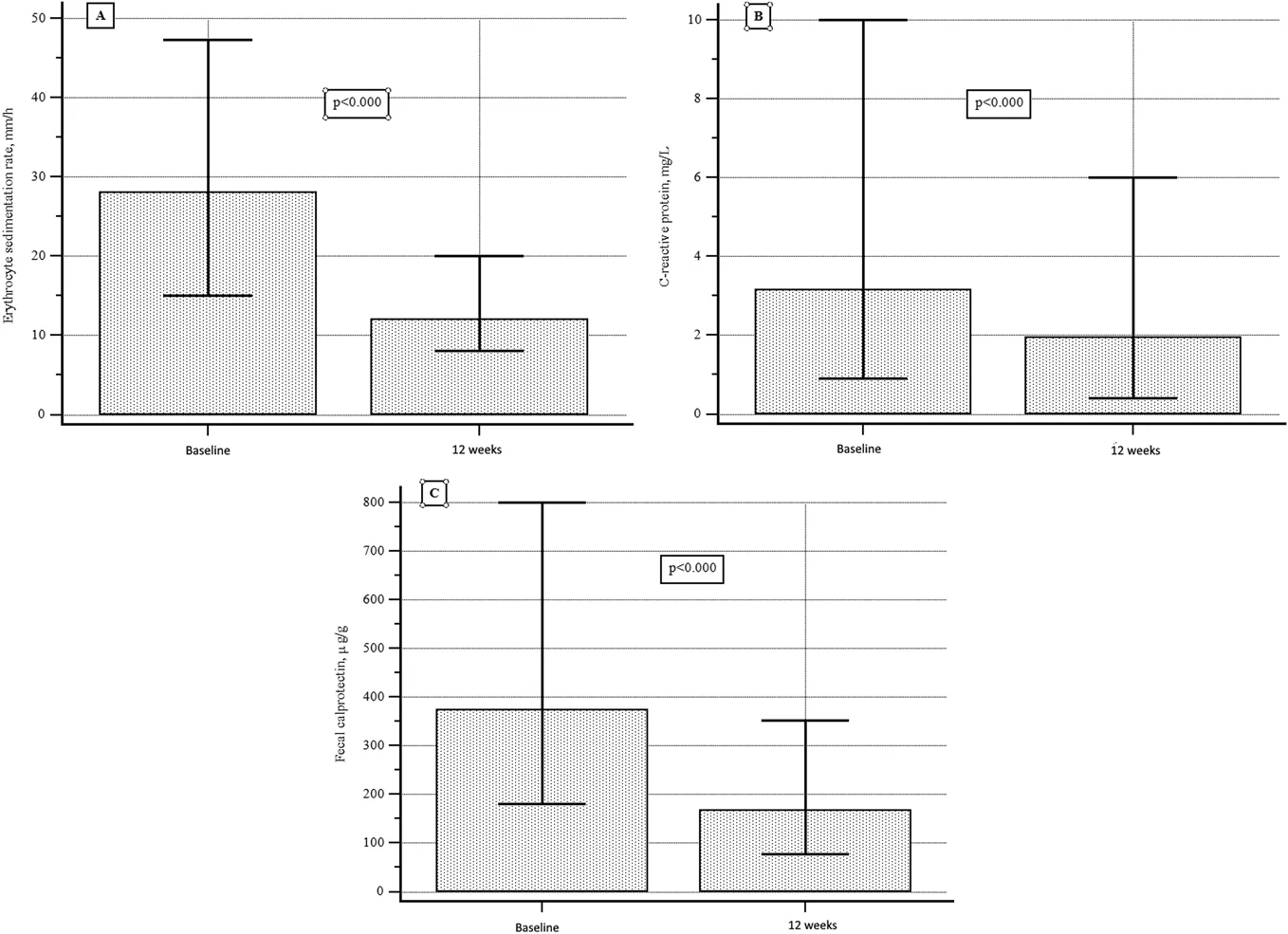
Laboratory results at the end of the induction period (12 weeks): C-reactive protein (panel A), Erythrocyte-sedimentation rate (panel B), and fecal calprotectin (panel C).

At baseline, median (IQR) ESR was 28.0 (15.0–46.5) mm/h, median (IQR) CRP was 3.0 mg/L (0.9–10.0), and median (IQR) FC was 363.0 μg/g (179.0–800.0) μg/g; at 12-week follow-up, ESR was 12.0 (8.0–20.0) mm/h, CRP was 2.0 mg/L (0.4–6.0) and FC was 168.5 μg/g (79.0–369.5) with a significant reduction compared with baseline (Figure 3A–C, respectively; *P* = 0.000).

## DISCUSSION

RW evidence is currently regarded as an essential tool, as it confers external validity to results from randomized controlled studies. Current RW studies have shown that RZB is effective in managing CD, particularly when used as second-line therapy (ie, after failure of anti-TNF-α agents). However, these studies are limited by small sample sizes or short follow-up.^15–22^

Our multicenter study relies on 1 of the largest cohorts of patients with CD for RW studies, currently published on the use of this anti-interleukin-23 agent. The first interesting finding of this study is that it confirmed the effectiveness of RZB in inducing remission in patients with CD who had failed advanced therapies. The CR rate in this study (76.4%) is consistent with that reported in most RW studies, which ranged from 48% to 85%.^15–22^ It is not easy to explain the vast differences in CR rates across studies. Characteristics of the enrolled population (ie, difference in disease extension, severity of the disease, or number of previous biologic treatments, etc.), the rate of concomitant medications, and the lower rate of well-recognized factors influencing the performance of the advanced therapies, such as smoking,^23^ could explain these differences in the results. Interestingly, the remission rates with and without steroids were very similar in our study, suggesting that RZB is effective and can achieve remission even without steroids. Moreover, we found that the sustained response to RZB was maintained for up to 52 weeks, indicating that the risk of loss of response during at least a 1-year follow-up is very low.

Another interesting result emerged when analyzing patients by line of treatment. We found that RBZ works better in non–advanced-therapy–naive patients with only 1 previous therapeutic failure, namely as a second-line therapy. This is a critical point to emphasize, because it suggests that early use of RZB in the pharmacological sequencing of CD may yield satisfactory outcomes. These results are likely linked to evidence that IL-23 is an essential driver of apoptosis evasion in response to anti-TNF in patients with CD, leading to resistance to anti-TNF-α through expansion of apoptosis-resistant T cells.^24^ However, we found that RZB also works well as a third-line or fourth-line treatment. Multifailure patients accounted for more than 40% of our population, and the 12-month RZB remission rate exceeded 70%, even among patients on fourth-line treatment. This means that RZB works well regardless of the treatment line, achieving consistently high remission rates.

The most interesting finding from the analysis of this population is that we identified factors that could negatively influence the attainment of remission. All the identified risk factors, such as age >40 years or the severity of endoscopic inflammation, could help us select patients with a higher probability of responding to RZB in the future. Notably, previous treatment with advanced therapies does not reduce the efficacy of RZB in multivariable analysis, even among patients who have already been treated with UST. Again, this means that RZB acts adequately regardless of the previous advanced therapy, achieving consistently high remission rates.

Looking at the coprimary end point, the rate of AEs was in line with the rates observed in the RW studies, which ranged from 3.8% to 29%.^15–22^ Most were mild and mainly characterized by respiratory tract infection. These types of AEs are similar to those reported in a recent postmarketing surveillance study.^25^ It is hypothesized that these AE occur because IL-23 is mainly involved in host protection against bacterial and parasitic infections,^26^ and IL-23 blocking could therefore limit the protection against the more common respiratory pathogens. Finally, the 2 cases of malignant neoplasms were recorded, but they do not seem to be related to the RZB treatment. On the contrary, in a large population of patients treated with multiple advanced therapies and followed for several months, the possibility of incidentally diagnosing a neoplasm could not be so rare.^16^

Looking at the secondary end points, interesting findings emerge. Overall, we found that RZB achieved mucosal healing in half of the treated patients, with significant reductions in inflammatory indices (CRP and FC) and a very low rate of CD-related surgical procedures. These findings are consistent with data from other RW studies^15–22^ and confirm the effectiveness of RZB.

Although this is 1 of the most extensive studies assessing the role of RZB in the RW management of CD, especially in patients with multiple failures of advanced therapies, it had several limitations that should be considered when interpreting the results. As a retrospective analysis, it is susceptible to recall bias and lacks the consistency afforded by standardized data collection protocols. The steroid tapering process was left to the investigators' discretion, potentially influencing the primary end point. In addition, longer follow-up was unavailable for most patients, and we cannot rule out the possibility that longer follow-up may have influenced the primary end point. Finally, endoscopic assessment was available in only a few patients, which may have limited the objective evaluation of RZB effectiveness. We know that the timing of the endoscopic reassessment should ideally be predefined at clear time points. However, this ideal end point could be used only for a prospective study. It is currently limited by 2 factors: first, follow-up endoscopies are still scheduled according to the physician's judgment based on patients' characteristics (clinical requirements, the severity of their disease, and how they respond to therapy)^27^; second, this is a retrospective study, in which the endoscopic time points were left to the real-life physician's discretion, in accordance with guidelines. Further, a well-designed prospective study should overcome this limitation of RW studies.

Despite these limitations, we believe the evidence from our study is sufficient to conclude that RZB is effective and safe for managing patients with CD after failure of a first-line advanced therapy, even in those with multiple previous failures. Of course, these results are not sufficient to provide clear guidance on the optimal positioning of RZB in the treatment of patients with CD. Important variables such as disease duration, disease location and behavior, presence of perianal disease, baseline disease severity, and previous exposure to advanced therapies may significantly influence treatment outcomes. They should be accounted for in future analyses to position RZB within the medical work-up of the CD clearly. Moreover, future studies should assess whether the long-term sustained response at 52 weeks is predictive of very long-term response, as with UST.^28^

## References

[1] Johnson AM Loftus EV, Jr. Risankizumab to treat moderately to severely active Crohn's disease in adults: an evaluation of trials and data. Expert Rev Gastroenterol Hepatol. 2023;17:1169–1183. 38095092 10.1080/17474124.2023.2295496

[2] Duerr RH Taylor KD Brant SR . A genome-wide association study identifies IL23R as an inflammatory bowel disease gene. Science. 2006;314:1461–1463. 17068223 10.1126/science.1135245 PMC4410764

[3] Gheita TA El Gazzar II El-Fishawy HS . Involvement of IL-23 in enteropathic arthritis patients with inflammatory bowel disease: preliminary results. Clin Rheumatol. 2014;33:713–717. 24384828 10.1007/s10067-013-2469-y

[4] Cua DJ Sherlock J Chen Y . Interleukin-23 rather than interleukin-12 is the critical cytokine for autoimmune inflammation of the brain. Nature. 2003;421:744–748. 12610626 10.1038/nature01355

[5] D'Haens G Panaccione R Baert F . Risankizumab as induction therapy for Crohn's disease: results from the phase 3 ADVANCE and MOTIVATE induction trials. Lancet. 2022;399:2015–2030. 35644154 10.1016/S0140-6736(22)00467-6

[6] Ferrante M Panaccione R Baert F . Risankizumab as maintenance therapy for moderately to severely active Crohn's disease: results from the multicentre, randomized, double-blind, placebo-controlled, withdrawal phase 3 FORTIFY maintenance trial. Lancet. 2022;399:2031–2046. 35644155 10.1016/S0140-6736(22)00466-4

[7] Peyrin-Biroulet L Chapman JC Colombel JF, SEQUENCE Study Group. Risankizumab versus ustekinumab for moderate-to-severe Crohn's disease. N Engl J Med. 2024;391:213–223. 39018531 10.1056/NEJMoa2314585

[8] Gomollón F Dignass A Annese V . 3rd European evidence-based consensus on the diagnosis and management of Crohn's Disease 2016: part 1: diagnosis and medical management. J Crohns Colitis. 2017;11:3–25. 27660341 10.1093/ecco-jcc/jjw168

[9] Lamb CA Kennedy NA Raine T . British Society of Gastroenterology Consensus Guidelines on the management of inflammatory bowel diseases in adults. Gut. 2019;68(suppl 3):S1–S106. 31562236 10.1136/gutjnl-2019-318484 PMC6872448

[10] Satsangi J Silverberg MS Vermeire S . The Montreal classification of inflammatory bowel disease: controversies, consensus, and implications. Gut. 2006;55:749–753. 16698746 10.1136/gut.2005.082909 PMC1856208

[11] Best WR. Predicting the Crohn's Disease activity index from the Harvey–Bradshaw Index. Inflamm Bowel Dis. 2006;12:304–310. 16633052 10.1097/01.MIB.0000215091.77492.2a

[12] Daperno M D'Haens G Van Assche G . Development and validation of a new, simplified endoscopic activity score for Crohn's disease: the SES-CD. Gastrointest Endosc. 2004;60:505–512. 15472670 10.1016/s0016-5107(04)01878-4

[13] Moskovitz DN Daperno M Van Assche G. Defining and validating cut-offs for the simple endoscopic score for Crohn's disease. Gastroenterology. 2007;132:S1097.

[14] Rutgeerts P Geboes K Vantrappen G . Predictability of the postoperative course of Crohn's disease. Gastroenterology. 1990;99:956–963. 2394349 10.1016/0016-5085(90)90613-6

[15] Alahmad M Quraishi MN Khan SW . Letter: early real-world evidence from the United Arab Emirates of the persistence, effectiveness and safety of risankizumab for the treatment of Crohn's disease. Aliment Pharmacol Ther. 2024;60:101–102. 38779929 10.1111/apt.18049

[16] Fumery M Caron B Hebuterne X . Long-term outcome of risankizumab in Crohn's disease: a real-world GETAID Study. Clin Gastroenterol Hepatol. 2024;22:2451–2458.e1. 38729389 10.1016/j.cgh.2024.04.016

[17] Zinger A Choi D Choi N . Risankizumab effectiveness and safety in Crohn's disease: real-world data from a large tertiary center. Clin Gastroenterol Hepatol. 2024;22:1336–1338.e2. 38065372 10.1016/j.cgh.2023.11.033

[18] Alsoud D Sabino J Franchimont D . Real-world effectiveness and safety of risankizumab in patients with moderate to severe multirefractory Crohn's disease: a Belgian multicentric cohort study. Inflamm Bowel Dis. 2024;30:2289–2296. 38215029 10.1093/ibd/izad315

[19] Zinger A Choi D Choi N . Long-term effectiveness and safety of risankizumab in patients with Crohn's Disease. Clin Gastroenterol Hepatol. 2025;23:1817–1823. 39461462 10.1016/j.cgh.2024.09.027

[20] Yarur AJ Ungaro R Huang K . Real-world effectiveness of ustekinumab in ulcerative colitis in a United States multicenter cohort consortium. Inflamm Bowel Dis. 2025;31:131–139. 38531068 10.1093/ibd/izae058 PMC11700893

[21] Macaluso FS Renna S Fries W . The effectiveness of risankizumab as induction therapy for Crohn's Disease: data from the Sicilian network for inflammatory bowel diseases. Inflamm Bowel Dis. 2025;31:1178–1181. 39460746 10.1093/ibd/izae248

[22] Johnson AM Askar M Belani S . A multicenter study of the real-world effectiveness and safety of risankizumab in Crohn's disease. J Crohns Colitis. 2025;19:jjaf070. 40289770 10.1093/ecco-jcc/jjaf070 PMC13032034

[23] Larsen MGR Overgaard SH Petersen SR . Effects of smoking on clinical treatment outcomes amongst patients with chronic inflammatory diseases initiating biologics: secondary analyses of the prospective BELIEVE cohort study. Scand J Immunol. 2024;100:e13395. 38973149 10.1111/sji.13395

[24] Atreya R Neurath MF. IL-23 blockade in anti-TNF refractory IBD: from mechanisms to clinical reality. J Crohns Colitis. 2022;16:ii54–ii63. 35553662 10.1093/ecco-jcc/jjac007 PMC9097672

[25] Shu Y Chen J Ding Y . Adverse events with risankizumab in the real world: postmarketing pharmacovigilance assessment of the FDA adverse event reporting system. Front Immunol. 2023;14:1169735. 37256136 10.3389/fimmu.2023.1169735 PMC10225532

[26] Frieder J Kivelevitch D Haugh I . Anti-IL-23 and anti-IL-17 biologic agents for the treatment of immune-mediated inflammatory conditions. Clin Pharmacol Ther. 2018;103:88–101. 28960267 10.1002/cpt.893

[27] St-Pierre J Rubin DT. Endoscopy in inflammatory bowel disease: indications, timing, and biopsy protocol. Gastrointest Endosc Clin N Am. 2025;35:1–18. 39510681 10.1016/j.giec.2024.04.001

[28] Mocci G Tursi A Scaldaferri F . Long-term effectiveness and safety of ustekinumab in Crohn's disease: results from a large real-life cohort study. J Clin Med. 2024;13:7192. 39685651 10.3390/jcm13237192 PMC11642252

